# The rate of X-ray-induced DNA double-strand break repair in the embryonic mouse brain is unaffected by exposure to 50 Hz magnetic fields

**DOI:** 10.3109/09553002.2015.1021963

**Published:** 2015-03-28

**Authors:** Lisa Woodbine, Jackie Haines, Margaret Coster, Lara Barazzuol, Elizabeth Ainsbury, Zenon Sienkiewicz, Penny Jeggo

**Affiliations:** ^a^ Genome Damage and Stability Centre, Life Sciences, University of Sussex, Brighton; ^b^ Public Health England, Centre for Radiation, Chemical and Environmental Hazards, Chilton, Oxfordshire, UK

**Keywords:** X-rays, extremely low frequency magnetic fields (ELF-MF), low dose radiation, DNA double-strand break repair, embryonic neuronal stem cells

## Abstract

*Purpose*: Following *in utero* exposure to low dose radiation (10–200 mGy), we recently observed a linear induction of DNA double-strand breaks (DSB) and activation of apoptosis in the embryonic neuronal stem/progenitor cell compartment. No significant induction of DSB or apoptosis was observed following exposure to magnetic fields (MF). In the present study, we exploited this *in vivo* system to examine whether exposure to MF before and after exposure to 100 mGy X-rays impacts upon DSB repair rates.

*Materials and methods*: 53BP1 foci were quantified following combined exposure to radiation and MF in the embryonic neuronal stem/progenitor cell compartment. Embryos were exposed *in utero* to 50 Hz MF at 300 μT for 3 h before and up to 9 h after exposure to 100 mGy X-rays. Controls included embryos exposed to MF or X-rays alone plus sham exposures.

*Results*: Exposure to MF before and after 100 mGy X-rays did not impact upon the rate of DSB repair in the embryonic neuronal stem cell compartment compared to repair rates following radiation exposure alone.

*Conclusions*: We conclude that in this sensitive system MF do not exert any significant level of DNA damage and do not impede the repair of X-ray induced damage.

## Introduction

We recently reported that X-ray-induced DNA damage, assessed by enumerating the DNA double-strand break (DSB) marker, 53BP1 foci, and the activation of apoptosis, can be sensitively quantified in the mouse embryonic neuronal stem and early progenitor cell compartment, called the ventricular and sub-ventricular zone (VZ/SVZ) (Gatz et al. 2011, Saha et al. 2014). From E11 to E16.5, the VZ/SVZ cells replicate rapidly and sensitively activate apoptosis (Pontious et al. 2008, Mitsuhashi and Takahashi 2009). Following *in utero* exposure at E13.5 to low radiation doses (10–100 mGy), we observed a linear induction of DSB and apoptosis in the VZ/SVZ with a statistically significant increase detectable after 10 mGy X-rays (Saha et al. 2014). This, therefore, represents a sensitive system to assess whether agents or exposures induce DNA damage.

Epidemiological studies have provided evidence for a statistically significant association between the risk of childhood leukaemia and prolonged exposure to elevated levels of power frequency magnetic fields (MF) or surrogates of MF exposure in the home (Greenland et al. 2000, Ahlbom et al. 2001, Draper et al. 2005, World Health Organization [WHO] 2007, Zhao et al. 2014). These findings suggest a potential doubling in risk with daily, mean exposures above 0.3–0.4 μT, with a possible effect at 0.2 μT. Hence, extremely low frequency MF (ELF-MF) have been classified as ‘possibly carcinogenic to humans’ (International Agency for Research on Cancer [IARC] 2002). Most carcinogens cause DNA damage and/or mutations. Multiple studies have examined whether ELF-MF exposure induces such genetic changes as chromosome aberrations, micronucleus formation, mutations and strand breaks. Although some positive findings have been obtained, the consensus for whether there is any impact remains controversial (IARC 2002, Ivancsits et al. 2003, Crumpton and Collins 2004, Mairs et al. 2007, Luukkonen et al. 2014). Many of the assays employed to monitor ELF-MF effects have restricted sensitivity since they use transformed cell lines, which have inherent genomic instability. Even cultured primary cell lines frequently have greater background damage than encountered *in vivo*. In our previous study, we therefore exploited our sensitive *in vivo* assay to assess DSB formation and apoptosis induction following exposure to 50 Hz fields at 100 or 300 μT (Saha et al. 2014). Despite detecting DSB and apoptosis after 10–25 mGy X-rays, we found no impact of ELF-MF and concluded that any DSB induction was less than that induced by 10 mGy X-rays, a useful comparison given that some computed tomography scanning procedures are equivalent to 10 mGy X-rays (Brenner and Hall 2007, Mettler et al. 2008).

In our previous work, we focused on DSB induction since DSB are significant for carcinogenesis. However, ELF-MF could potentially have impacts on the DNA damage response distinct to inducing DSB. A recent study found that pre-exposure to 50 Hz MF modified the genotoxic effects caused by exposure to menadione (Luukkonen et al. 2011). Further, other studies have reported that ELF-MF exposure can potentiate the mutagenicity, level of strand breaks or micronuclei induced by ionizing radiation (Lagroye and Poncy 1997, Ding et al. 2000, Miyakoshi et al. 2000, Mairs et al. 2007). As a potential explanation, we considered that any single strand break (SSB) or base damage arising from ELF-MF exposure could potentially enhance the persistence of DSB either by promoting their formation following replication and/or by impeding DSB repair. Of relevance in this context, MF exposure affects the kinetics of radical pair reactions, potentially causing the release of reactive oxygen species (ROS) (Brocklehurst and McLauchlan 1996, O’Dea et al. 2005). Interestingly, the sensitivity of radical repair reactions to external MF has been proposed to underlie magnetoreception in migratory birds, and to modulate enzymatic reactions involving radical pairs (Maeda et al. 2012, Fedele et al. 2014). Thus, either a change in the level of oxidative damage or modulation of the efficacy of the DSB repair machinery could explain the above findings. Given these previous reports of a potentiating effect of ELF-MF exposure on the response to exposure to X-rays or menadione, here, we extended our previous analysis, which examined simply whether ELF-MF exposure induced DSB, to examine if these fields can enhance or impede the repair of radiation-induced DSB. We did not observe any influence of ELF-MF on the rate of DSB repair following 100 mGy X-rays.

## Materials and methods

### Mice

Time-mated pregnant female C57BL/6 mice were obtained from the MRC Mary Lyon Centre (Harwell, Oxfordshire, UK) on E5.5–6.5. Except for assay optimization, all animal exposures and embryonic head preparations were undertaken at Public Health England (PHE), Chilton, Oxfordshire, UK.

### Exposure to X-rays or 50 Hz magnetic fields

Animals were exposed to X-rays on E13.5, using an A.G.O. HS X-ray System, model CP160/1 (Ago X-Ray Ltd, Martock, Somerset, UK) at 250 kV constant potential with a compound filter of copper and aluminium (X-ray spectrum with a half-value layer of 2 mm of copper) at a dose rate equal to 4.9 mGy min^21^; a 100 mGy exposure thus took 20 min. Groups of 1–4 mice were irradiated in a Correx^®^ polythene filtered box (Williton Box Co., Taunton, Somerset, UK). Following irradiation, animals were returned to the stock holding room. Sham exposures were treated in the same way as exposed animals, although for operational reasons the animals were placed for 10 min within the X-ray system.

**Figure 1.**
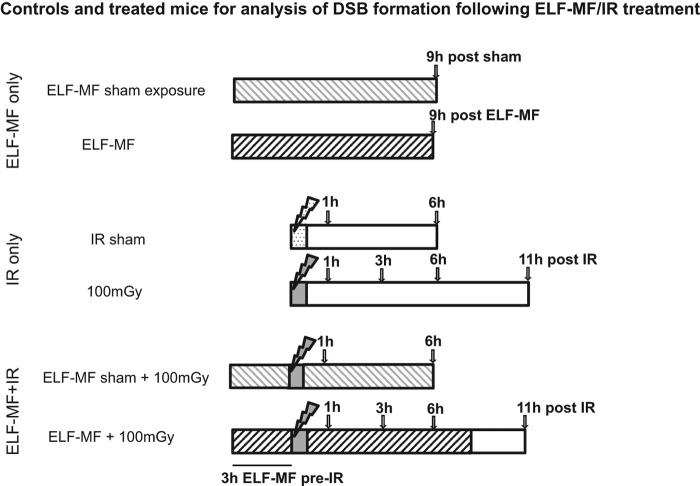
Plan of treatments and controls used for analysis of 50 Hz magnetic field, 100 mGy IR or combined exposures. For sham or ELF-MF exposure alone (lines 1–2), mothers were sham exposed (light grey shading) or exposed at 300 μT (dark grey shading) in the Helmholtz coils for 9 h, and samples collected as indicated by arrows. For sham or IR exposure alone (lines 3–4), mothers were sham exposed (dotted light grey) or exposed (dark grey) in the IR facility, and samples collected at 1 and 6 h post sham, or at 1, 3, 6 and 11 h post IR. For combined exposed to ELF-MF and IR, mothers were sham-exposed to ELF-MF for 3 h, exposed to IR at 100 mGy, then sham exposed to ELF-MF post IR, with samples collected at 1 or 6 h (line 5); or exposed to ELF-MF at 300 μT for 3 h, exposed to IR at 100 mGy, then exposed to ELF-MF at 300 μT up to 9 h post IR, with samples collected at 1, 3, 6 and 11 h (line 6).

The MF exposure system was as described previously (Kabacik et al. 2013, Saha et al. 2014). Briefly, sinusoidal MF were generated using a function generator and power amplifier connected to a pair of Helmholtz coils (inner radius of 25.5 cm). During exposure (or sham exposure), up to four pregnant mice were housed in a non-metallic cage in the centre of the coils. Animals were provided with standard diet (SDS RM3; Lillico, Kent, UK) and water, and were observed using a CCTV system. Animals were exposed to a vertical, 50 Hz MF at 300 μT for times indicated in Figure 1 on E13.5. At all times, the variation in the field was less than 5% of the nominal. Following MF exposure with or without X-ray exposure, animals remained in the cage within the coils until the end of the experiment. During all exposures, the static MF was maintained at 43 μT, the average static field in the laboratory containing the exposure system. Timed sham exposures (no current through the coils) were run on consecutive days. The average background 50 Hz MF in the laboratory housing the exposure system (measured over 24 h using an EMDEX II MF dosimeter) was less than 0.1 μT; average noise levels were 63 dB(A) and ambient light levels were 60 ± 5 lux. All experiments complied with the Animals (Scientific Procedures) Act 1986 (UK) and were approved by the local Animal Welfare and Ethical Review Board.

### Embryonic head preparation

1, 3, 6, 9 or 11 h after X-irradiation and/or ELF-MF exposure, the pregnant animals were sacrificed by cervical dislocation and foetuses immediately removed. The head of each foetus was detached from the body, washed in pre-cooled PBS and embedded using RA Lamb-OCT cryo-embedding compound (Thermo Fisher Scientific, Loughborough, Leicestershire, UK). The heads were snap frozen in liquid nitrogen-cooled isopentane (VWR, Lutterworth, Leicestershire, UK) and stored at − 70°C prior to cryo-sectioning. The total time for this process was approximately 15 min. They were transported overnight to the University of Sussex in dry ice.

### Immunohistology

Cryosectioning (sagittal, 7 μm) and 53BP1 staining was as previously described (Saha et al. 2014). All analysis was carried out using a Nikon eclipse microscope and conducted blindly. Embryos were categorized into groups so that complete coverage of exposures (ionizing radiation [IR], MF or controls) underwent analysis within a close time frame.

### 53BP1 foci analysis and quantification

53BP1 foci were quantified in a delineated region representing ∼ half the VZ/SVZ most distal from the ventricle as previously described (Saha et al. 2014). Two sections were quantified for each embryo with a minimum of 100 cells (regardless of DSB numbers) being quantified per section.

### Statistical analysis

Data analysis was carried out using Minitab^®^ version 15. The Anderson Darling Normality testing was applied to check adherence to the Normal distribution. General linear model analysis of variance (ANOVA) was applied to compare the full set of experimental factors of repair time (1, 3, 6, 11 h); mother (up to 4, nested in repair time); X-ray exposure (0, 100 mGy), and ELF-MF exposure (0, 300 μT) as well as to directly compare the numbers of foci in each experimental exposure group (X-ray sham, 100 mGy X-rays, ELF-MF sham, ELF-MF 300 μT, ELF-MF sham + 100 mGy, ELF-MF 300 μT + 100 mGy X-rays) at each repair time. Pairwise comparisons (Tukey's test) were also used to compare foci numbers and exposure categories at each repair time-point. Levene's test was used to test for equality of variances across the exposure categories in the full experimental model.

## Results

### DSB repair analysis

The embryonic VZ/SVZ houses the neuronal stem and early progenitor cells (Bayer et al. 1991, Pontious et al. 2008, Mitsuhashi and Takahashi 2009). From E11 to E16.5, the VZ/SVZ cells proliferate rapidly and at E13.5 the VZ/SVZ represents the predominant region between the lateral ventricle and cortical plate (Cox et al. 2006, Saha et al. 2014). For comparison with our previous findings, we exposed pregnant mice to ELF-MF and/or X-rays at E13.5. The enumeration of γH2AX foci is commonly used to quantify DSB formation and repair. However, pan nuclear staining of γH2AX can also rise in replicating cells. We observed a background of γH2AX staining in the VZ/SVZ at E13.5 due to the substantial level of replication, which diminished the sensitivity for detecting low levels of DSB (detectable as more defined foci above the pan nuclear background staining) (Gatz et al. 2011). To enhance sensitivity, we enumerated 53BP1, another marker of DSB, rather than γH2AX foci, since 53BP1 foci do not form during replication. Previously, we observed low 53BP1 foci numbers without irradiation with a dose-dependent increase following radiation exposure (Gatz et al. 2011, Saha et al. 2014). To assess the rate of DSB repair, pregnant mothers were exposed to 100 mGy X-rays at E13.5 and 53BP1 foci enumerated at 1, 3, 6, and 11 h post exposure (Figure 1). To assess the influence of ELF-MF on the rate of DSB repair, pregnant mothers were exposed to 50 Hz fields (300 μT continuously) 3 h prior to X-irradiation and for 1, 3, 6 or 9 h post X-irradiation (for the latter, samples were collected 11 h after X-irradiation; Figure 1). The intensity of the ELF-MF used here was the highest flux density used in our previous study. Control treatments (Figure 1) include ELF-MF exposure or sham treatment for 9 h, both collected alongside the 6 h post IR samples (a full time course for sham exposure was not included since we did not previously detect any biological impact). 53BP1 foci levels at 1 h post IR were taken to represent the induction level. For each group, three embryos from each of four mothers were examined.

### Exposure to ELF-MF did not influence the repair of X-ray-induced DSB

**Figure 2.**
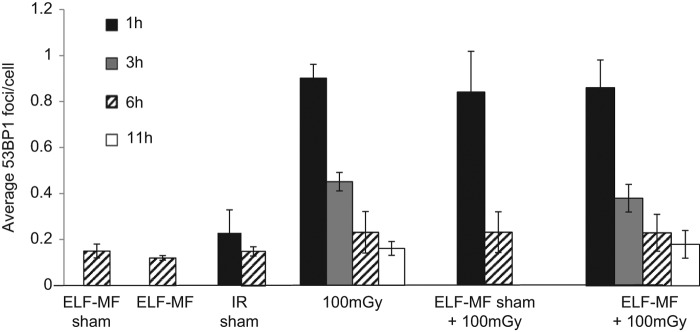
Analysis of 53BP1 foci formation and disappearance after *in utero* exposure of embryos to ELF-MF at 300 μT, 100 mGy IR, or combined exposures. 53BP1 foci were quantified at 1, 3, 6, or 11 h post IR in the VZ/SVZ following sham exposure, exposure to 50 Hz ELF-MF at 300 μT, 100 mGy IR or both. Embryos (E13.5) were exposed *in utero*. Results represent the mean of 2 sections from 3 embryos from each of 4 mothers (i.e., a sample size of 24). Only two mothers were examined for IR sham (i.e., a sample size of 12). Error bars are standard deviation of repeat measurements (at least 2 animals per group).

Sham-irradiated embryos had 0.1–0.2 53BP1 foci per cell and exposure to 100 mGy X-rays induced ∼ 0.8–0.9 DSB/cell (at 1 h post IR) demonstrating, as expected, that 100 mGy X-rays causes a statistically significant increase in 53BP1 foci (Figure 2; ANOVA *p* for X-ray exposure in the full experimental model, < 0.001). Repair time was also found to be a significant experimental factor (*p* < 0.001). In contrast, there was no evidence of a significant influence of ELF-MF exposure (ANOVA *p* for full experimental model = 0.957; ANOVA between groups for sham vs. ELF-MF exposure *p* = 0.995), nor was there any evidence of ELF-MF exposure impacting on repair time (ANOVA full experimental model, interaction: ELF-MF × time, *p* = 0.679).

Background and X-ray-induced foci counts were slightly greater than our previous findings of ∼ 0.1 53BP1 foci/cell (background) and 0.6 53BP1/cell (after 100 mGy X-rays). We attributed this slightly enhanced sensitivity to the current 53BP1 antibodies, which appeared slightly more sensitive than those used previously. Following irradiation, either with or without ELF-MF exposure, DSB are > 50% repaired by 3 h and background foci levels are almost reached by 6 h (pairwise comparisons 1 and 3 h, and 1 and 6 h, *p* both < 0.001). Foci levels at 11 h post IR were slightly lower but the difference was not statistically significant (pairwise comparison 6 and 11 h, *p* = 0.182; note that irradiated samples exposed to sham ELF-MF treatment at 3 or 11 h post exposure was not examined). As observed previously there was variation between mothers. However, this factor was only just statistically significant (ANOVA *p* for the full experimental model = 0.042). Additionally, ANOVA means that the variance of each factor is apportioned separately and there was no evidence of inequality of variances across the exposure categories (i.e., it is unlikely that there were other unidentified interaction effects; Levene's test *p* = 0.638). Therefore this result does not impact on the overall significance of the repair time and X-irradiation, or the lack of significance of the ELF-MF or ELF-MF-repair time interaction.

ANOVA comparing the experimental exposure categories at each repair time revealed no difference between sham X-irradiated, sham ELF-MF, or ELF-MF exposure groups (pairwise comparison *p* values all > 0.995) at 1 h; no difference between X-irradiated, X-irradiated + sham ELF-MF or X-irradiated + ELF-MF exposure groups (pairwise comparison *p* values all > 0.914) at 1 h, and no differences between any of the exposure categories at 3, 6 or 11 h; further evidencing a lack of effect of ELF-MF exposure on repair time. In particular, it should be noted that there was no significant difference between the X-ray sham and 300 μT ELF-MF treatments (Tukey's simultaneous pairwise comparison *p* > 0.999) or the 100 mGy X-ray and 300 μT + 100 mGy treatment groups (Tukey's pairwise *p* = 1.000) and there was no evidence of any interaction effects.

## Discussion

The embryonic VZ/SVZ represents a sensitive tissue to examine DSB formation following exposure to low dose IR as well as ELF-MF (Gatz et al. 2011, Saha et al. 2014). Here, we monitored DSB repair rates following IR exposure in the embryonic VZ/SVZ and observed a rate similar to that observed in non-replicating mouse embryonic fibroblasts (Nijnik et al. 2007). We then assessed how exposure to ELF-MF before and after exposure to 100 mGy IR influences the rate of repair of the radiation-induced DSB. At E13.5, the VZ/SVZ cells are rapidly replicating and increased formation of base damage or DNA SSB is likely to affect DSB levels, either directly by impeding DSB repair, or indirectly by causing DSB following replication of SSB/base damage. No evidence for lack of normality was identified, therefore full General Linear model ANOVA was applied to elucidate the effect of each experimental factor (Mother, 100 mGy X-ray exposure, 300 μT ELF-MF exposure) on foci numbers at 1–11 h post X-rays, and to search for evidence of interaction between ELF-MF and repair time. No evidence for interaction was observed. We stress also that after 100 mGy IR, ∼ 1% of the cells undergo apoptosis (and this frequency was not altered by ELF-MF exposure, data not shown). After 100 mGy nearly all cells harbour a DSB. Thus, the assessment of DSB induction and repair is not influenced to any significant degree by ongoing apoptosis.

The failure to find any impact of combined ELF-MF and IR exposure in this sensitive *in vivo* stem cell system contrasts to the findings of some studies using cultured cell lines, where exposure to ELF-MF has been shown to have a potentiating impact on IR and other DNA damaging agents (Lagroye and Poncy 1997, Ding et al. 2000, Miyakoshi et al. 2000, Mairs et al. 2007). Several of these studies used tumor cell lines (e.g., human glioma cells) and very high magnetic flux densities (> 1 mT). It is possible that changes in tumor cells enhance any impact of ELF-MF. Indeed, in one study, the impact of ELF-EMF exposure on X-ray induced mutations was only observed in NF-kappa B-inhibited cells (Ding et al. 2000). However, these studies also frequently involved very long exposures (< 24 h) to ELF-MF, and it is possible that the duration of exposure is critical and that longer exposure times might increase the likelihood of observing an impact. An interesting recent study observed that 24-h pretreatment with ELF-MF increased the level of DNA damage monitored by the comet assay as well as micronucleus formation of subsequent treatment with menadione, a radical-inducing agent (Luukkonen et al. 2011). This study used 100 μT, which is a lower flux density than employed here. However, this study used neuroblastoma cells and menadione treatment, which is very distinct to radiation.

In summary, although our findings do not eliminate the possibility that ELF-MF might induce some level of SSB/base damage which can impact under certain situations, when combined with our previous study, they consolidate the evidence that low level ELF-MF exposure does not induce DNA damage able to cause a biological impact in this sensitive *in vivo* system, which monitors the response of stem cells.
